# Long non-coding RNAs in ovarian cancer

**DOI:** 10.1186/s13046-018-0793-4

**Published:** 2018-06-19

**Authors:** Lei Zhan, Jun Li, Bing Wei

**Affiliations:** 1 Department of gynecology and obstetrics, The Second Affiliated Hospital of Anhui Medical University, Hefei, 230601 China; 2 School of Pharmacy, Anhui Key Laboratory of Bioactivity of Natural Products, Anhui Medical University, Hefei, 230032 China

**Keywords:** Long non-coding RNAs, Ovarian cancer, Dysregulated expression, Clinical implications, Well characterized mechanism

## Abstract

Long non-coding RNAs (lncRNAs) refer to functional cellular RNAs molecules longer than 200 nucleotides in length. Unlike microRNAs, which have been widely studied, little is known about the enigmatic role of lncRNAs. However, lncRNAs have motivated extensively attention in the past few years and are emerging as potentially important regulators in pathological processes, including in cancer. We now understand that lncRNAs play role in cancer through their interactions with DNA, protein, and RNA in many instances. Moreover, accumulating evidence has recognized that large classes of lncRNAs are functional for ovarian cancer. Nevertheless, the biological phenomena and molecular mechanisms of lncRNAs in ovarian cancer remain to be better identified. In this review, we outline the dysregulated expression of lncRNAs and their potential clinical implications in ovarian cancer, with a particular emphasis on discussing the well characterized mechanisms underlying lncRNAs in ovarian cancer.

## Background

Cancers of the ovary, cervix and uterus are the common types of gynecological oncology. Ovarian cancer is the most lethal gynecological cancer in women globally. Of which, epithelial ovarian cancer (EOC) is the most common group of ovarian cancers. Based on histopathology, immunohistochemistry and molecular genetic analysis, EOC mainly includes endometrioid, clear-cell, mucinous, high-grade and low-grade serous carcinomas. These types of tumors are inherently different diseases and can be reproducibly diagnosed by light microscopy [1]. It was estimated that there are 52,100 new cases and 22,500 deaths from ovarian cancer in 2015 in China [2]. The American Cancer Society estimates that there will be 22,440 new cases and 14,080 deaths from ovarian cancer in 2017 in the United States [3]. Despite recent improvements in cytoreductive surgery and chemotherapy, the 5-year survival rate of ovarian cancer is still approximately 30–40% owing to its late diagnosis and the chemoresistance [4, 5]. Thus, it is becoming essential to explored novel avenues to fight against the deadly cancer. In the past few years, a growing body of researches illustrated an intriguing emergent correlation between lncRNAs and ovarian cancer which may offer novel biomarkers for ovarian cancer prognosis or diagnosis.

With the advance of high-resolution microarray and massively parallel sequencing technology [6, 7], it has been well accepted that more than 98% of the human genome is transcribed into RNA transcripts that do not have apparent protein-coding potential. These noncoding RNAs (ncRNAs) can be classified into lncRNAs (> 200 nucleotides) and small ncRNAs (≤200 nucleotides) according to their size [8]. Small ncRNAs such as microRNAs (miRNAs), small interfering RNAs (siRNAs) and PIWI-interacting RNAs (piRNAs) have been intensely investigated for many years because of their remarkable functions on a plethora of diseases initiation and progression [9–11]. However, lncRNAs are a relatively poorly understood class of ncRNAs. The most agreeable definition of lncRNAs is the tautological one: lncRNAs are long RNA transcripts that do not encode proteins. Nevertheless, lncRNAs expressions are even more tightly regulated than that of protein-coding genes. According to the latest statistics from Encyclopedia of DNA Elements (GENCODE) database, there are 15,941 lncRNA genes in 2015 compared with 6496 lncRNA genes in 2009, showing a great rise in the number of lncRNAs in less than a decade and the total number of lncRNAs still continues to climb, which makes lncRNAs earned an enormous amount of attention in the past few years [12, 13]. However, only few of them have been functionally characterized. It has been reported that lncRNAs exert widely biological functions across every branch of life, such as evolutionary conservation, imprinting genomic loci, chromatin remodeling, regulating enzymatic activity allosterically and modulate different cell fates [14–17]. Now, it is becoming clear that lncRNAs have a prominent function in ovarian cancer, the over-expression, deficiency or mutation of lncRNAs are associated with tumorigenesis, metastasis, prognosis or diagnosis. In this review, we first provide an overview of lncRNAs biology and then describe dysregulated expression of lncRNAs and their potential clinical implications in ovarian cancer. Finally, we make a special effort to investigate the well characterized mechanisms underlying lncRNAs in ovarian cancer and summarize future challenges in the field.

### Overview of lncRNAs

LncRNAs were first found in the early 1990s [18], but they were really identified as a class of RNA molecules until in 2002 [19]. Currently, lncRNAs are primarily categorized based on their genomic proximity between neighboring transcripts and which can be categorized as sense (sense intronic or overlapping a protein-coding gene), antisense, the second most prevalent group in humans (overlapping one or more exons of another transcript on the same or opposite strand), bidirectional promoter (transcribed within 1 kb of promoters antisense to the protein-coding transcript), intronic (transcribed from an intron of a protein-coding gene), intergenic, the largest subclass of lncRNA molecules in humans (lies as an independent unit within the genomic interval between two protein-coding genes), enhancer (transcribed from an enhancer region of a protein-coding gene) and two unique lncRNAs, circular RNAs (act as miRNA sponges and sequester miRNAs) [8, 13, 20] and sno-lncRNAs (processed from excised and debranched introns by exonucleolytic trimming and carry out their functions in complex with specific protein components by forming ribonucleoprotein complexes) [21].

To date, although only a small number of functional lncRNAs have been well characterized, while a number of examples of biologically functional lncRNAs have already been reported. LncRNAs have been demonstrated to affect several important physiological processes by interacting with DNA, chromatin, signaling, regulatory proteins and a variety of cellular RNA species. Chromatin-bound lncRNAs can regulate gene expression by controlling local chromatin architecture or directing the recruitment of regulatory molecules to specific loci. LncRNAs interaction with multiple proteins can promote the assembly of protein complexes or impair protein-protein interactions, mRNA interactions with lncRNAs can recruit protein machinery involved in multiple aspects of mRNA metabolism to affect splicing, mRNA stability, or translation or sequester miRNA away from target mRNA [22–27]. Whereupon, the lncRNAs also can be classified into four categories according to their mechanism of action, including signals (act as a molecular marker for functional biological conditions in a spatiotemporal manner, responding to developmental cues, cellular signals and other stimuli) [28], decoys (repress transcription by interfering or competing with other RNAs or proteins that bind to DNA) [29], guides (bind to proteins and transport the complex to chromatin and other specific targets through direct or indirect interaction to the DNA, leading to changes in the gene expression of either neighboring or distantly located genes) [30] and scaffolds (serve as central platforms on which different effector molecules are assembled. The assembled complexes can design strategies to selectively utilize specific signaling components and outputs to initiate the corresponding biological functions, such as transcriptional repression or activation) [31].

### LncRNAs and ovarian cancer

#### Dysregulated expression of lncRNAs and their potential clinical implications in ovarian cancer (Table 1)

***HOX genes*** HOX gene clusters are well known to regulate the embryo body plan and contribute to cell specification in several adult differentiation processes by encoding an evolutionary conserved family of transcription factors. There are A, B, C and D HOX genes located on four different chromosomes in humans. The HOX genes produce hundreds of lncRNAs that show similar spatiotemporal windows of expression to their neighboring protein-coding genes [32]. Hox transcript antisense intergenic RNA (HOTAIR) is the best described lncRNA in HOX clusters [33]. The HOXA genes determine the identity of body segments and reported to be abnormally expressed in cancers. Protein coding genes are located on the sense strand of the HOXA gene clusters, while non-coding genes are located on the antisense strand (AS). The 5-prime region of HOXA includes three lncRNAs, HOTTIP, HOXA11-AS, and HOXA10-AS [34–36] and studies found that HOTAIR and HOXA11-AS were dysregulated in ovarian cancer.

**Table 1:** Dysregulated expression of lncRNAs and their potential clinical implications in ovarian cancer

LncRNA	Differential expression in ovarian carcinoma	Clinical application	References
HOTAIR^a^	Up	HOTAIR levels were positively correlated with the FIGO stage, histological grade of the tumor, lymph node metastasis. HOTAIR was an independent prognostic factor for OS and DFS in ovarian cancer patients	[37, 38]
HOXA11-AS^a^	Down in EOC	High HOXA11-AS in SOC expression in tumor tissues was an independent predictor of poor PFS and OS	[40, 41]
	Up in SOC		
Xist^a^	Down	Reduced expression of Xist in patient samples was correlated with a shorter progression-free interval	[43–45]
ANRIL^a^	Up	Elevated ANRIL expression was positive correlated with advanced FIGO stage, high histological grade, lymph node metastasis. ANRIL was an independent prognostic factor for OS.	[54, 55]
MEG3^a^	Down	No evidence	[61, 62]
HOST2^a^	Up	No evidence	[64]
FAL1^a^	Up	FAL1 occurred frequently in epithelial cancer and were associated with clinical outcomes in patients with ovarian cancer	[65]
LSINCT5^a^	Up	No evidence	[66]
ZNF300P1^a^	Down	No evidence	[67]
TC1500845, TC0901107	Up	No evidence	[68]
TC0100223, TC0101686	Down	No evidence	
TC0101441^a^	Up	TC0101441 was an independent prognostic factor for OS in ERα-positive ovarian cancer	
AB073614^a^	Up	5-year OS in ovarian cancer patients with high expression of AB073614 was inferior to that with low expression	[73]
ASAP1-IT1	Up	High expression of ASAP1-IT1 and FAM215A were associated with favorable OS	[74]
FAM215A	Up		
PVT1^a^	Up	Low expression levels of PVT1 and lnc-SERTAD2–3 exhibited longer PFS and OS. PVT1, lnc-SERTAD2–3 and hsa-miR-200c-3p simultaneously can stratify patients’ risk of relapse into three discrete classes, high risk, mixed and low risk	[60]
lnc-SERTAD2–3	Up		
RP11-284 N8.3.1	Down	RP11-284 N8.3.1 and AC104699.1.1 significantly divide stages III and IV ovarian cancer patients into high- and low-risk groups and their increased expressions were associated with a decreased risk of survival	[77]
AC104699.1.1	Down		
AC005562.1, AC105760.2, EPB41L4A-AS1, MCM3AP-AS1	Up	Potential candidates associated with stage progression in ovarian cancer and could classify patients into high-and low-risk subgroups with significantly different survival outcomes	[81]
AC074117.10, MEG8, RP11-220I1.1, RP11-429 J17.2, RP11-618G20.1	Down		
AC092214.10, CYP3A5, LEMD1, PART1, RNF157-AS1, RP11-532F12.5	Up	No evidence	[83]
AC010680.1, ADAMTS9-AS1, ADAMTS9-AS2, AK021537, AK125532, GRTP1-AS1, LEMD1-AS1, LOC386758, LOC729970, RP1–7814.1, RP11-597D13.9, LEMD1-AS1	Down		
lnc-SOX4–1, lnc-HRCT1–1	Up	Low expression levels of lnc-SOX4–1 and lnc-HRCT1–1 exhibited longer PFS and OS	[60]
RUNX1-IT1, MALAT1	Up	RUNX1-IT1, MALAT1, H19, HOTAIRM1, LOC100190986 and AL132709.8 were associated with OS and DFS	[49]
H19^a^	Majority expressed in benign, borderline and invasive SOC, but not in epithelial cells of the mucinous tumors		
HOTAIRM1, LOC100190986, AL132709.8	Up		
SPRY4-IT1	Up	High lncRNA SPRY4-IT1 expression is an independent prognostic factor for PFS and OS	[85]
BC037530, AK021924, AK094536, AK094536, BC062365	Up	Higher expressions of lncRNAs BC037530, AK021924, AK094536, AK094536, BC062365 and lower expressions of lncRNAs BC004123 BC007937 were associated with ovarian cancer patients survival and can serve as an independent predictor apart from FIGO stage and patient grade	[86]
BC004123, BC007937	Down		
LOC100288181	Up	High Lnc-OC1 expression was associated with poor prognosis of ovarian cancer patients	[87]
DUXAP10	Up	The overall survival of patients with ovarian cancer whose DUXAP10 was overexpressed was significantly lower than that in the DUXAP10 low expression group. Tumor stage in DUXAP10 high expression group was higher, and the tumor volume was larger.	[88]

^a^ mechanism described in ovarian cancer

HOTAIR is an lncRNA transcribed from the HOXC locus, it represses transcription by recruiting polycomb repressive complex 2 (PRC2) [33]. The role of HOTAIR in ovarian cancer was widely researched in recent years. HOTAIR was shown to be elevated in EOC tissues and serous ovarian cancer (SOC) tissues. HOTAIR levels were positively correlated with the FIGO stage, histological grade of the tumor, lymph node metastasis. Multivariate analysis suggested that HOTAIR was an independent prognostic factor for predicting overall survival (OS) and disease-free survival (DFS) in EOC and SOC patients [37, 38].

HOXA11-AS is an antisense transcript lncRNA of the HOX11 gene [39]. HOXA11-AS expression levels were significantly lower in human EOC tumors than normal ovarian tissues [40]. Of note, however, in SOC tissues, HOXA11-AS was found to be up-regulated, higher HOXA11-AS expression was significantly correlated with histological grade and preoperative CA125. Multivariate Cox regression analysis revealed that high HOXA11-AS expression in tumor tissues was an independent predictor of poor progression-free survival (PFS) and OS [41]. The opposite levels of HOXA11-AS in EOC and SOC need to be investigated in future studies.

### Xist

LncRNA Xist regards as a lncRNA that only transcript expressed exclusively from the inactive X chromosome [42]. Several studies suggested that Xist was lost or down-regulated in ovarian cancer cell lines or recurrent ovarian cancer cell lines [43, 44]. Furthermore, down-regulated Xist in ovarian cancer showed shorter PFS [45]. Loss of Xist seemed related with breast cancer-associated gene 1 (BRCA1) mutation in ovarian cancer, but the conclusion remained controversial, which will be discussed in the below in the manuscript.

### H19

Actually, the first discovered abnormal expression lncRNA in ovarian cancer was the imprinted maternally expressed H19 gene [46]. H19 was the first identified imprinted lncRNAs which is maternally expressed only and critical for the maintenance of its opposite imprinted gene insulin-like growth factor 2 (Igf2) which from the paternal allele [47]. Tanos et al. studied the expression of H19 in epithelial ovarian cancer and found H19 was expressed in the majority of benign, borderline and invasive serous carcinomas, but no expression of H19 was seen in the epithelial cells of the mucinous tumors [48]. Recent study investigated that H19 was associated with OS and DFS in ovarian cancer [49]. In patients with epithelial ovarian cancer, loss of heterozygosity of both Igf2 and H19 genes tend to be found in advanced clinical stages of ovarian cancer, loss of imprinting of Igf2 and H19 genes may be contributed to the development of ovarian cancer [50]. The later study showed that loss of imprinting of Igf2 was not a prominent mechanism for Igf2 over-expression in serous epithelial tumors, but high frequency of epigenetic alterations at the Igf2/H19 domain was actually more frequently observed in benign tumors, indicating that epigenetic changes of Igf2/H19 may be an early indicator of ovarian cancer [51]. These results suggest that the establishment of primary imprints on different genes might be mechanistically linked, even when they are oppositely imprinted.

### ANRIL

The antisense non-coding RNA in the INK4 locus (ANRIL, also known as CDKN2B-AS), which encoded in the chromosome 9p21 region, is transcribed as a 3.8-kb lncRNA and strongly implicates in the epigenetic regulation of INK4b/ARF/INK4a gene cluster by binding PRC1 and PRC2 [52, 53]. Recent studies delineated that ANRIL could be a potential prognostic biomarker in EOC and SOC. The following examples conform to this opinion. A 2015 study showed that ANRIL was over-expressed in SOC tissues and highly metastatic SOC cell lines. Elevated ANRIL expression was positive correlated with advanced FIGO stage, high histological grade, lymph node metastasis. Univariate log-rank tests and multivariate Cox regression analyses revealed that patients with high ANRIL expression exhibited poor OS [54]. The later research in 2016 reported that ANRIL expression was significantly elevated in EOC tissues compared with noncancerous tissues, increased ANRIL level was correlated with advanced FIGO stage, high histological grade and poor OS [55].

### PVT1

Amplification of genes on chromosome 8q24 is a common event in serous ovarian cancer, which always associated with reduced survival duration [56, 57]. Using genome copy number and transcriptional analyses, PVT1 and the oncogene MYC were well illustrated to be amplified on chromosome 8q24 [58, 59]. Recent study identified that low expression levels of PVT1 and lnc-SERTAD2–3 exhibited longer PFS and OS. PVT1, lnc-SERTAD2–3 and hsa-miR-200c-3p simultaneously can stratify patients’ risk of relapse into three discrete classes, high risk, mixed and low risk [60].

### MEG3

Maternally expressed gene 3 (MEG3), which locates at the chromosome 14q32 locus, is a long non-coding RNA that can inhibit tumorigenesis and progression of various types of cancers is also involved in ovarian cancer [61, 62].

### HOST2

Human ovarian cancer-specific transcript 2 (HOST2) was a novel member of the HOST family of long non-coding mRNA-like gene [63]. The level of HOST2 was significant up-regulated in EOC-derived cell line and clinical EOC samples compared to control groups [64].

### FAL1

In a genome-wide survey on somatic copy-number alterations (SCNAs) of lncRNA in 2394 tumor specimens from 12 cancer types in combination with bioinformatics analyses of lncRNA SCNAs and expression with functional screening assays, Hu et al. [65] found an oncogenic lncRNA, focally amplified lncRNA on chromosome 1 (FAL1) occurred frequently in epithelial cancer and were associated with clinical outcomes in patients with ovarian cancer.

### LSINCT5

A novel lncRNA, named LSINCT5, was characterized as a 2.6 Kb polyadenylated, long stress-induced non-coding transcript that is on the negative strand. LSINCT5 localized in the nucleus and potentially transcribed by RNA polymerase III. Silva and co-workers [66] first found LSINCT5 was over-expressed in breast and ovarian cancer cell lines and tumor tissues, relative to their normal counterpart.

### ZNF300P1

Using sequenom massARRAY methylation analysis, Gloss and colleagues identified a novel epigenetic regulated lncRNA ZNF300P1 (also named LOC134466), was hypermethylated in 81% of Type II EOC and could differentiate tumours from normal ovarian surface epithelial cells, indicating that methylation of ZNF300P1 may act as a diagnostic biomarker for EOC [67].

### Microarray analyses identified lncRNAs

Using microarray analysis Qiu et al. identified 51 lncRNAs were up-regulated and 64 lncRNAs were down-regulated following E2 treatment in E2 receptor alpha (ERα)-positive EOC SKOV3 cells. Further analysis by qRT-PCR revealed that the expression of TC1500845, TC0101441 and TC0901107 was significantly up-regulated by E2, whereas TC0100223 and TC0101686 were significantly down-regulated by E2. Furthermore, multivariate analysis indicated that TC0101441 was an independent prognostic factor for OS ERα-positive ovarian cancer [68].

Human metastasis-associated lung adenocarcinoma transcript 1 (MALAT1) is a newly identified metastasis-associated lncRNA [69, 70]. In the microarray analysis of lncRNAs, MALAT1 was found to be significantly increased in ovarian cancer tissues and different ovarian cancer cell lines [71, 72]. Furthermore, multivariate analysis indicated that MALAT1 was an independent predictor of survival and Kaplan-Meier analysis revealed that patients with increased MALAT1 expression had a poorer DFS time [72].

Arraytools (http://linus.nci.nih.gov/BRB-ArrayTools) found lncRNA AB073614 was consistently up-regulated in ovarian cancer tissue compared to the normal tissue. Kaplan-Meier analysis and log-rank test revealed that the 5-year OS in ovarian cancer patients with high expression of AB073614 was inferior to that with low expression [73].

In a study including 266 EOC patients, Fu and colleagues identified high expression of ASAP1-IT1, FAM215A and LINC00472 were more frequently in low grade tumors and early stage disease compared to high grade tumors and late stage disease respectively in primary EOC. High expression of ASAP1-IT1 and FAM215A were associated with favorable OS [74].

LncRNAs have a penchant to be co-expressed with their neighboring coding-genes [75]. RNA-sequence analyses can be used to obtain whole-transcriptome shotgun sequences and detect the less-abundant mRNA and lncRNA transcripts. However, RNA-sequence was not perfect in detecting gene expression when two genes were close to each in the genome. To test this effect, “networks analysis” were applied to determine the potential roles of differentially expressed lncRNAs and the correlation between the differentially expressed lncRNAs and mRNAs in cancer [76].

Guo et al. used a multi-step approach to construct a functional lncRNA-mRNA regulatory network (OVLMN) and investigated the co-expression relationships between the differentially expressed lncRNAs and coding genes. Here, 295 lncRNAs and 2366 mRNAs were identified to be significantly co-expressed lncRNA-mRNA pairs. Two hub lncRNAs, RP11-284 N8.3.1 and AC104699.1.1, which were one-step neighbors in the OVLMN, could significantly divide stages III and IV ovarian cancer patients into high- and low-risk groups and their increased expressions were associated with a decreased risk of survival. Furthermore, these two lncRNAs RP11-284 N8.3.1 and AC104699.1.1 played protective role throughout malignant ovarian cancer progression which were associated with the activation of the immune system and anti-tumor processes in the ovarian cancer microenvironment [77].

It has been shown that diverse RNA molecules which harboring miRNA response elements (MREs) can act as competing endogenous RNAs (ceRNAs) [78]. CeRNA crosstalk represents a novel cluster of miRNA regulatory network and forms miRNA-mediated ceRNA networks (ceRNETs). Naturally, perturbation of ceRNA crosstalk perturbation will disrupt the balance of the ceRNETs and leading to disease initiation and progression [79, 80]. Recently, lncRNA-associated ceRNETs in ovarian cancer was proposed by Zhou and co-workers for the first time [81]. These authors validated interaction network among miRNAs, mRNAs and lncRNAs in 401 OvCa patients from Human 1.0 ST array from the Cancer Genome Atlas (TCGA) data portal (http://cancergenome.nih.gov/) [82] and 1270 miRNA-mediated ceRNA crosstalk between lncRNAs and mRNAs (LMceCTs) were identified. Furthermore, it was found that these mRNAs were significantly enriched in six functional clusters including RNA splicing, biosynthetic process, cell death and apoptosis, cell cycle, morphogenesis and development and mRNA catabolic process, and several pathways including mTOR signaling pathway, TGF-β signaling pathway, insulin signaling pathway, VEGF signaling pathway and p53 signaling pathway, which are well known to contribute to the pathogenesis of ovarian cancer. Using ceRNA-network driven method investigated ten lncRNA ceRNAs (four risky lncRNAs AC005562.1, AC105760.2, EPB41L4A-AS1, MCM3AP-AS1 and six protective lncRNAs AC074117.10, MALAT1, MEG8, RP11-220I1.1, RP11-429 J17.2, RP11-618G20.1) were identified as potential candidates associated with stage progression in OvCa and the ten lncRNAs could classify patients into high-and low-risk subgroups with significantly different survival outcomes.

Wang and colleagues performed microarray analyses to identify dysregulated lncRNAs in malignant EOC. In total, they identified 182 up-regulated and 481 downreguated transcripts of lncRNAsin in malignant EOC compared with the benign cyst as well as the normal ovary groups. Using qPCR to validate the microarray data and indicated that AC092214.10, CYP3A5, LEMD1, PART1, RNF157-AS1 and RP11-532F12.5 levels increased, whereas AC010680.1, ADAMTS9-AS1, ADAMTS9-AS2, AK021537, AK125532, GRTP1-AS1, LEMD1-AS1, LOC386758, LOC729970, RP1–7814.1, RP11-597D13.9 and LEMD1-AS1 levels decreased. Networks analyses found the neighboring coding gene functions of up-regulated lncRNAs were primarily involved in the mitotic cell cycle, mitotic nuclear division, and the cell cycle, the neighboring coding gene function of the down-regulated lncRNAs included cell metabolic process, organic substance metabolic process, and gene expression. Pathway analysis revealed that p53 signaling pathway, protein processing in endoplasmic reticulum, the TGF-β signaling pathway, and the MAPK signaling pathway are engaged in the pathogenic process of malignant EOC. LncRNA and mRNA co-expression networks determined that two antisense lncRNAs (RP11-597D13.9 and ADAMTS9-AS1) were associated with their nearby coding genes FAM198B, ADAMTS9 respectively, which participated in cancer progression. This study is the first report on lncRNA expression profiling in the normal ovary, benign cysts and malignant EOC [83].

A microarray study of lncRNA expression in 5 well-known ovarian cancer cell lines (OVCAR8, OVCAR3, A2780, OVCA432 and CAOV3) identified more than 5000 lncRNAs were association with OS and PFS, of which low expression levels of lnc-SERTAD2–3, lnc-SOX4–1, lnc-HRCT1–1 and plasmacytoma variant translocation gene (PVT1) exhibited longer PFS and OS by using univariate and multivariate analysis. Network analysis [84] identified PVT1 and lnc-SOX4–1 were highly inter-connected, high levels of PVT1 or lnc-SOX4–1 in patient with poor prognosis presented altered PIK3/AKT and MAPK signaling pathways which were related to cell cycle, proliferation, anti-apoptosis, inflammation and metastasis [60].

Lately, six lncRNAs RUNX1-IT1, MALAT1, H19, HOTAIRM1, LOC100190986 and AL132709.8 were validated to be associated with recurrence of ovarian cancer through using the microarray data preprocessing. These six lncRNAs were independent of OS and DPS according to multivariate and sub-group analyses. Networks analyses indicated that these six lncRNAs were involved in cancer-related biological processes and pathways [49].

From the above stated, lncRNAs could be biomarkers and have potential clinical implications in ovarian cancer. A lot of recent researches alo have illustrated the potential of lncRNAs as diagnostic or prognostic biomarkers in ovarian cancer. LncRNA sprouty RTK signaling antagonist 4 (SPRY4)-IT1 expression was significantly up-regulated in ovarian tumor tissues and ovarian cancer cell lines in comparison with adjacent non-tumor control tissues and the human ovarian immortalized nontumorigenic ovarian surface epithelial, respectively. Kaplan-Meier survival analysis and multivariate analysis indicated that high lncRNA SPRY4-IT1 expression may be an independent prognostic factor for PFS and OS in ovarian cancer patients [85]. Zhan et al. identified higher expressions of lncRNAs BC037530, AK021924, AK094536, AK094536, BC062365 and lower expressions of lncRNAs BC004123 BC007937 were associated with ovarian cancer patients survival and can serve as an independent predictor apart from FIGO stage and patient age [86]. LOC100288181 (also named as Lnc-OC1) was significantly up-regulated in ovarian cancer tissues and Kaplan-Meier survival analysis confirmed that high Lnc-OC1 expression was associated with poor prognosis of ovarian cancer patients [87]. The expression of lncRNA DUXAP10 in ovarian cancer tissues was significantly higher than that in normal ovarian tissues. Clinical data analysis showed that the overall survival of patients with ovarian cancer whose DUXAP10 was overexpressed was significantly lower than that in the DUXAP10 low expression group. Furthermore, tumor stage in DUXAP10 high expression group was higher, and the tumor volume was larger [88].

### Well characterized mechanisms underlying lncRNAs in ovarian cancer (Fig. 1)

#### Increasing cell migration and invasion

It was indicated that silencing of HOTAIR suppressed EOC migration and invasion in vitro and in vivo [37]. Furthermore, MMP3, MMP9 and EMT-related genes (E-cadherin, Vimentin, and Snail) were the downstream mediators of HOTAIR activity affecting EOC cell migration and invasion [37].

**Fig. 1:**
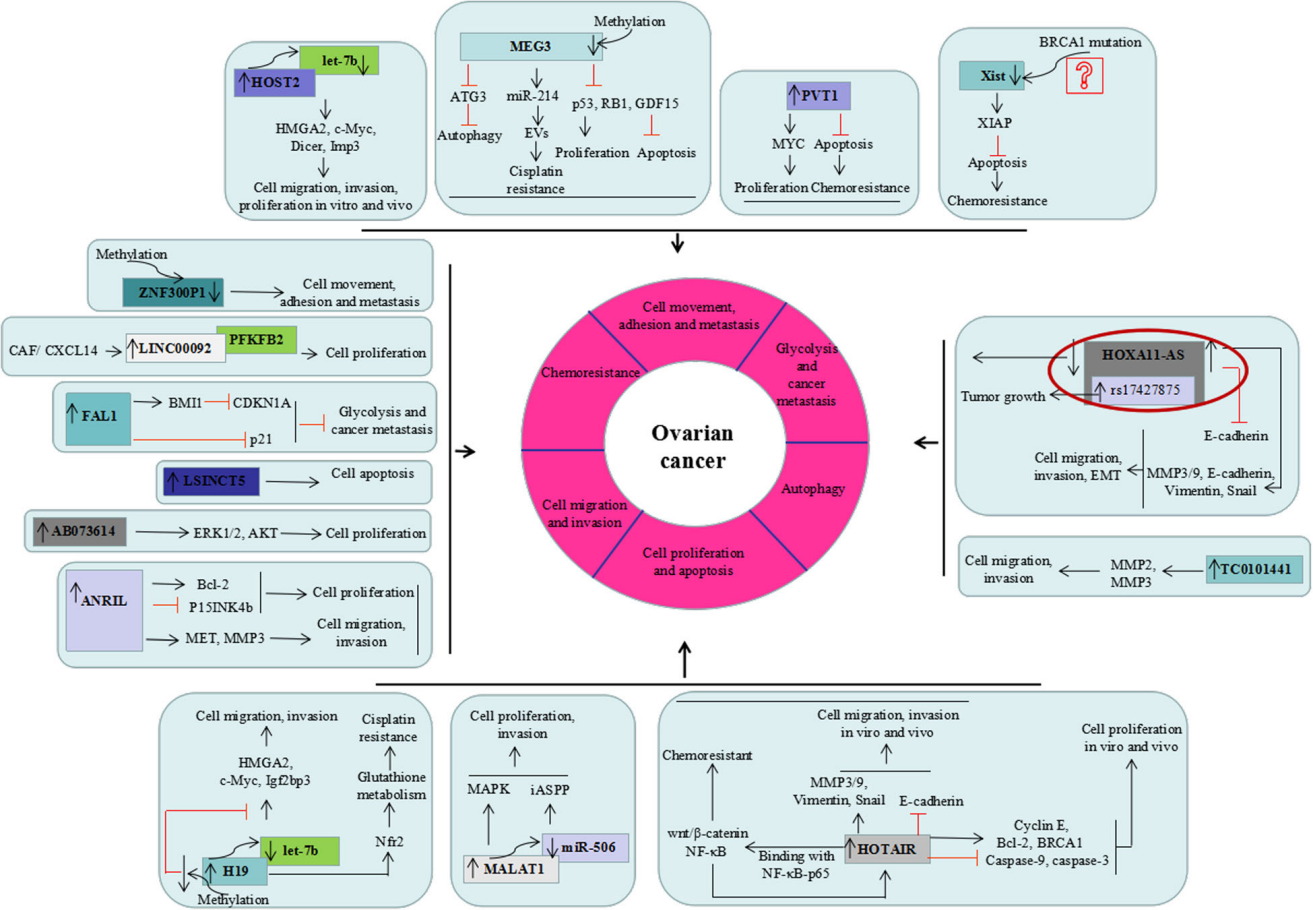
Well characterized mechanisms underlying lncRNAs in ovarian cancer. The mechanism mainly including increasing cell migration and invasion, increasing cell proliferation and inhibiting cell apoptosis, inducing chemoresistant, promoting cell movement, adhesion and metastasis, increasing glycolysis and cancer metastas and inhibiting autophagy Of special note is the role of HOXA11-AS in ovarian cancer owing to its levels were significantly lower in human EOC tumors but were up-regulated in SOC tissues compared to normal ovarian tissues

LncRNA HOXA11-AS rs17427875, which is a novel functional variant in HOXA11-AS. In vitro study of ectopic expression of HOXA11-AS rs17427875 in EOC cells showed decreased survival, migration, and invasion compared to common allele A expression. The finding was supported by xenograft experiments in which stable expression of HOXA11-AS rs17427875 reduced primary tumor growth to a greater extent than common allele A in vivo [40]. Mechanistically, silencing of HOXA11-AS in SOC cell line led to cell proliferation inhibition, thereby restricting cell invasion and migration by down-regulating MMP, VEGF and EMT marker genes levels [89].

H19 expression was showed to be regulated by DNA methylation, methylation of H19 contributed to ovarian cancer inhibition. For example, metformin and histone H1.3 inhibited ovarian cancer cell migration and invasion by down-regulating H19 via DNA methylation [90–92]. Furthermore, H19 depletion impaired the motility and invasiveness of tumor cells was part through inhibiting let-7-mediated regulation of metastasis-promoting genes, including Hmga2, c-Myc and Igf2bp3 [90]. Additionally, recent report was indicating that H19 may be predictive of responsiveness to ovarian cancer therapy. For instance, in a preliminary experiment by Mizrahi and colleagues developed a new therapy strategy to target the expression of plasmid expressing diphtheria toxin A chain (DTA) gene under the control of H19 regulatory sequences in ovarian carcinoma cell lines and in a heterotopic animal model for ovarian cancer, in which intraperitoneal administration of DTA-H19 (The H19 plasmid was digested with Xba I and Nco I and the insert of the luciferase gene was replaced by the DTA coding region to yield the DTA-H19 construct) has the potential to kill ascites tumor cells, deliver its intracellular toxin without targeting normal tissues [93]. This pioneering study is the first in the ovarian cancer ascites fluid to demonstrate the therapeutic potential of modulating H19 in vivo. Whether this initial intriguing finding can be translated into a treatment for solid tumors remains to be determined.

It has been suggested that that the functions of some lncRNAs might be attributed to miRNAs [25]. miRNA let-7b, was well known as a potent tumor suppressor [94], was reported to acted as a candidate regulator for lncRNA HOST2 [64]. In vitro and in mice experiment showed that silencing of HOST2 expression inhibited cell migration, invasion and proliferation in EOC. Bioinformatics analysis indicated that HOST2 harbored a let-7b binding site and binding with let-7b, further promoted the endogenous expression of metastasis-promoting genes including HMGA2, c-Myc, Dicer and Imp3 that were targeted by let-7b [64].

Mechanistic investigations in vitro validated that silencing of ANRIL could attenuate cell migration and invasion of SOC by inhibiting MET and MMP3 expression [54]. Furthermore, knockdown of TC0101441 suppressed E2-induced cell migration and invasion by inhibiting MMP2 and MMP3 [95].

### Increasing cell proliferation and inhibiting cell apoptosis

In SOC, Knockdown of HOTAIR inhibited A2780 and OVCA429 cells proliferation in vitro and impaired tumor growth in vivo. Mechanistic research demonstrated that HOTAIR silencing retarded SOC cell cycle progression and promoted cell apoptosis by decreasing cyclin E, histone H1 (a histone mark associated with cyclin E), anti-apoptotic protein Bcl-2, tumour suppressor gene BRCA1 expression and increasing pro-apoptotic proteins caspase-9 and caspase-3 expression, which were related with cell cycle and apoptosis [38].

Two novel mechanisms underlying MALAT1 involved in ovarian cancer were identified. MALAT1 promoted ovarian cancer cell proliferation and invasion via activating MAPK pathways [96]. Gain-of-function and loss-of-function analyses suggested that MALAT1 acted as an oncogenic lncRNA via miR-506-dependent iASPP regulation [97].

PVT1 was suggested as a MYC activator [98]. Investigation of the mechanism of silencing of PVT1 or MYC in ovarian and breast cancer cell lines demonstrated reduced levels of PVT1 or MYC inhibited cell proliferation, reduced levels of PVT1 but not MYC increased cell apoptosis, indicating that increased expression of both MYC and PVT1 contributed to the pathophysiology of ovarian cancer and breast cancer pathophysiology; however, PVT1 acted independently of MYC in generation of the apoptotic phenotype [58].

Mechanistic investigations in A2780 cells suggested that FAL1 play the oncogenic activity by stabilizing the epigenetic repressor BMI1, which is a subunit of PRC1 [99], further inhibited their common target gene CDKN1A expression, which is well known to be involved in cell-cycle arrest and apoptosis. In addition, FAL1 also exerted its oncogenic function via suppression of p21 expression. Of special note is that FAL1 siRNA was injected into the peritoneal cavity of an orthotopic mouse model for late-stage ovarian cancer caused inhibition of tumor growth, indicating that targeting FAL1 may represent an approach in ovarian cancer treatment [65].

The expression of MEG3 was down-regulated in EOC tissues and cell lines due to its promoter methlytion. In vitro study indicated that over-expression of MEG3 in OVCAR3 cells inhibited proliferation and promoted apoptosis by promoting p53, GDF15 and RB1 expression [100]. Furthermore, it was suggested that loss of LSINCT5 function inhibited proliferation in breast and ovarian cancer cell lines [66]. Mechanistically, silencing of AB073614 inhibited ovarian cancer cells proliferation in vivo and in vitro through partial suppressing ERK1/2 and AKT-mediated signaling pathway [73]. Gain-of-function and loss-of-function in combination with in vivo analyses demonstrated that ANRIL promoted EOC cell proliferation by up-regulating Bcl-2 and down-regulating P15^INK4b^ [55]. In addition, silencing H19 induced apoptosis through mitochondrial and caspases dependent pathway in ovarian cancer cell lines [101].

### Inducing chemoresistant

Acquisition of drug resistance is one of the main obstacles encountered in cancer chemotherapy [102]. HOTAIR has been demonstrated to significantly influence chemotherapy in ovarian cancer, indicating that HOTAIR could be a candidate prognostic marker in ovarian cancer patients during chemotherapy. For example, in a study based on 1080 human ovarian cancer samples offered solid evidence that HOTAIR and its surrogate DNA methylation signature were significantly positive correlation with both risk of relapse and of death in the carboplatin-treated patients. Notably, these researches challenged the dogma that cisplatin and carboplatin have the same effect on ovarian cancer with the findings HOTAIR and its surrogate DNA methylation signature were associated only with carboplatin resistance but not cisplatin resistance. They preliminarily speculated the differential response of carboplatin and cisplatin is due to underlying differences in how the mesenchymal stem cells (MSC) biology of tumour stroma and cell membrane transport characteristics affect the two drugs [103]. To determine the underlying functional mechanism of HOTAIR over-expression and its role in chemoresistant in ovarian cancer, gain-of-function and loss-of-function study in combination with in vivo in nude mouse model analyses demonstrated that HOTAIR induced cellular resistance to cisplatin was through activating the wnt/β-catenin pathway [104]. A recent study by Nephew and co-workers suggested that platinum-induced DNA damage contributed to HOTAIR activation of the NF-κB pathway and cellular senescence. Furthermore, DNA damage response activated-NF-κB induced HOTAIR and formed a positive-feedback loop, resulting in sustained NF-κB activation and persistent DNA damage signaling. Chromatin immunoprecipitation assay in A2780-CR5 cells investigated the underlying mechanism of HOTAIR in activation NF-κB/DNA damage was through the binding between HOTAIR promoter region and NF-κB-p65 [105].

BRCA1 is a well-characterized breast and ovarian tumor suppressor, females with mutations in BRCA1 are predisposed to develop breast and ovarian cancers [106, 107]. Chromatin immunoprecipitation investigated that BRCA1 associated with Xist and colocalized with the Xist RNA-coated chromatin of the inactive X chromosome (Xi) in ovarian cancer cells. Mutation of BRCA1 led to loss of Xist and marked defects in Xi chromatin structure [108, 109]. However, Xiao et al. was contentious and reported that Xist RNA localization or X-linked gene expression in female somatic cells was independent on mutation or depletion of BRCA1 because Xist transcript remained abundant and can coat the Xi despite significant depletion of BRCA1. BRCA1 reconstitution in HCC1937 cells did not rescue a localization defect for Xist RNA [110]. The charm of the results by Silver’s and Xiao’s researches raise the interesting that communication between BRCA1 and Xist-coated Xi may be a reflection of a larger role for BRCA1 in maintaining heterochromatin structure or function. Of note, Mechanistic investigations found that down-regulation of Xist might increase the expression level of X-linked inhibitor of apoptosis (XIAP) and block Taxol-induced apoptosis to cause resistance phenotype, suggesting that Xist may be a potential marker for chemotherapeutic responses in ovarian cancer [44].

Mechanistically, in a recent study revealed a close link between H19 and glutathione metabolism in the regulation of cancer-drug resistance in high-grade SOC. Up-regulated H19 in cisplatin resistance ovarian cancer cell lines promoted Nrf2 and its target genes expression which involved in glutathione metabolism transcription and glutathione increase, further caused cisplatin inactivation and reduced free radicals, resulting in cisplatin resistance in vitro [111].

A recent study revealed that demethylation of MEG3 by curcumin and 5-AZA-dC treatment in EOC cell lines weakened the extracellular vesicles’ (EVs) capability of enhancing cisplatin resistance via binding with miR-214 [112]. Furthermore, increased expression of PVT1 and its negative function in apoptosis caused cisplatin or carboplatin-docetaxel resistance in ovarian cancer [113, 114].

### Promoting cell movement, adhesion and metastasis

To further investigate the function of ZNF300P1 in ovarian cancer, the researchers applied loss-of-function study and showed that ZNF300P1 knockdown resulted in activation of cellular movement, promotion of cell adhesion and metastasis, as well as loss of cellular polarity in type II epithelial ovarian cancer [115].

### Increasing glycolysis and cancer metastasis

As is shown in a recent study, lncRNA microarray analysis found LINC00092 was induced upon stimulation by cancer-associated fibroblasts (CAF)-secreted CXCL14 in A2780s cells. Gain-of-function and loss-of-function in combination with in vivo analyses established that LINC00092 promoted ovarian cancer metastasis by binding with a glycolytic enzyme, 6-phosphofructo-2-kinase/fructose-2, 6-biphosphatase 2 (PFKFB2), which proved to be critical for ovarian cancer metastasis and glycolytic phenotype, indicating that LINC00092 acted in ovarian cancer-associated fibroblasts to drive glycolysis and progression of ovarian cancer [116].

### Inhibiting autophagy

A novel regulatory mechanism of autophagy by MEG3 was shown in EOC. Up-regulation of MEG3 by transfecting with MEG3 or treatment with actinomycin D inhibited progression of EOC by targeting ATG3 and inducing autophagy. This study is the first to show that lncRNAs affect autophagy in ovarian cancer [117].

## Conclusions and future challenges

The transcriptional landscape of all organisms is far more complex than was originally imagined, as the vast majority of genomic sequence is pervasively transcribed into a diverse range of protein-coding RNAs and ncRNAs. Traditionally, a number of genome’s repertoire of non-protein-coding transcripts may be viewed as inconsequential transcriptional “noise” or “garbage”. In this expanded view of both the genome and the transcriptome, our catalogue of genetic elements is now brimming with lncRNAs. The lncRNAs studies have gradually become one of the most noticeable parts in the field of RNA biology. In recent years, a large number of lncRNAs have been identified and there is an exponential growth of studies on the biological functions of lncRNAs in human cancers, including ovarian cancer. The functional roles and mechanisms of action of some classically defined lncRNAs are well studied in ovarian cancer, and this list of characterized lncRNAs continues to grow. High-throughput sequencing technologies reveal numerous novel lncRNAs transcripts that play pivotal roles in ovarian cancer. Networks analysis is applied to determine the potential roles of differentially expressed lncRNAs and the correlation between the differentially expressed lncRNAs and mRNAs in ovarian cancer. Most important, some specific lncRNAs have the potential to be translated into clinical implications for diagnosis, prognosis of ovarian cancer.

However, the study of lncRNAs in ovarian cancer comes up some deficiencies. First, with current deep RNA-sequencing and advanced epigenomic technologies, the rate of discovering new lncRNA genes is rapidly outpacing the rate of characterizing them, only a few lncRNAs have been well characterized in ovarian cancer and even very few lncRNAs can used for clinical implications. Furthermore, most of the current studies in ovarian cancer-associated lncRNAs are focused on the role of lncRNAs on cell fates. However, structural approaches [118] to evaluating lncRNAs functions which can reveal the functionally relevant sites involved in miRNA or protein binding are limited. Last, it has showed that lncRNAs expressed in a cell type-, tissue-, developmental stage-or disease state-specific manner [119]; in this review, we found the lncRNAs HOXA11-AS expression levels were significantly lower in human EOC tumors but were up-regulated in SOC tissues compared to normal ovarian tissues, in view of the contradictorily results, we think, the study of lncRNAs in ovarian cancer should be exact to specific type of ovarian cancer. We hope the continuous development of a toolkit for studying lncRNAs offers promise for the elucidation of lncRNAs functions and for the regulation of lncRNAs expression for therapeutic purposes in ovarian cancer.

## Abbreviations

ANRIL: Antisense non-coding RNA in the INK4 locus
AS: Antisense strand
BRCA1: Breast cancer-associated gene 1
CAF: Cancer-associated fibroblasts
ceRNAs: Competing endogenous RNAs
ceRNETs: CeRNA networks
DFS: Disease-free survival
DTA: Diphtheria toxin A chain
EOC: Epithelial ovarian cancer
ER: E2 receptor
Evs: Extracellular vesicles’
FAL1: Focally amplified lncRNA on chromosome 1
GENCODE: Encyclopedia of DNA Elements
HOST2: Human ovarian cancer-specific transcript 2
HOTAIR: Hox transcript antisense intergenic RNA
Igf2: Insulin-like growth factor 2
LmceCTs: LncRNAs and mRNAs
LncRNAs: Long non-coding RNAs
MALAT1: Metastasis-associated lung adenocarcinoma transcript 1
MEG3: Maternally expressed gene 3
miRNAs: MicroRNAs
MREs: MiRNA response elements
MSC: Mesenchymal stem cells
OS: Overall survival
PFKFB2: 6-phosphofructo-2-kinase/fructose-2, 6-biphosphatase 2
PFS: Progression-free survival
piRNAs: PIWI-interacting RNAs
PRC2: Polycomb repressive complex 2
PVT1: Plasmacytoma variant translocation gene
SCNAs: Somatic copy-number alterations
siRNAs: Small interfering RNAs
SOC: Serous ovarian cancer
SPRY4: Sprouty RTK signaling antagonist 4
Xi: Inactive X chromosome
XIAP: X-linked inhibitor of apoptosis
